# Supplementary material for: A high-resolution mRNA expression time course of embryonic development in zebrafish
Source: eLife. 2017 Nov 16;6:e30860. doi: 10.7554/eLife.30860 (PMC5690287; doi:10.7554/eLife.30860)
Supplement: Supplementary file 6. [file elife-30860-supp6.zip › biolayout-clusters-files/Cluster206-genes.html]

Cluster206


# Cluster206: Genes

| Ensembl ID | Gene Name | Chr | Start | End | Biotype |
| --- | --- | --- | --- | --- | --- |
| ENSDARG00000041170 | aagab | 18 | 19823542 | 19830746 | protein\_coding |
| ENSDARG00000101130 | adck4 | 15 | 14618341 | 14653720 | protein\_coding |
| ENSDARG00000062370 | bcl2l13 | 18 | 20592619 | 20619234 | protein\_coding |
| ENSDARG00000058148 | dnajc7 | 3 | 17728441 | 17759394 | protein\_coding |
| ENSDARG00000054224 | ipo11 | 8 | 32019492 | 32345450 | protein\_coding |
| ENSDARG00000003167 | mlst8 | 12 | 18421909 | 18438665 | protein\_coding |
| ENSDARG00000011496 | ppm1bb | 12 | 25341266 | 25393949 | protein\_coding |
